# Antimicrobial Systems Based on Essential Oils Advanced by Zinc Oxide Nanoparticles

**DOI:** 10.1021/acsptsci.6c00106

**Published:** 2026-05-19

**Authors:** Greta Kaspute, Urte Prentice, Roman Viter, Arunas Ramanavicius

**Affiliations:** † Department of Nanotechnology, State Research Institute Center for Physical Sciences and Technology (FTMC), Sauletekio Av. 3, LT-10257 Vilnius, Lithuania; ‡ Department of Personalised Medicine, State Research Institute Centre for Innovative Medicine, Santariskiu St. 5, LT-08410 Vilnius, Lithuania; § Department of Mechatronics, Robotics and Digital Manufacturing, Faculty of Mechanics, Vilnius Gediminas Technical University, Plytines St. 25, LT-10105 Vilnius, Lithuania; ∥ Latvia Faculty of Exact Sciences and Technologies, University of Latvia, Jelgavas street 3, Riga LV-1004, Latvia; ⊥ Department of Physical Chemistry, Institute of Chemistry, Faculty of Chemistry and Geosciences, Vilnius University, Naugarduko St. 24, LT-03225 Vilnius, Lithuania

**Keywords:** ZnO nanoparticles, essential oils, advanced systems, antibacterial effects, advanced materials, plants

## Abstract

Zinc oxide nanoparticles (ZnO NPs) have emerged as a promising material to enhance the performance of essential oils (EOs) in various applications due to their unique properties, including chemical stability, biocompatibility, and antimicrobial efficacy. This review examines the potential of combining ZnO NPs with EOs, focusing on their integration into polymeric matrices for improved antimicrobial effectiveness, stability, and controlled release. The incorporation of ZnO NPs with EOs shows significant promise in fields such as food packaging, medicine, and agriculture, offering a natural alternative to synthetic chemicals. The review addresses key challenges like antimicrobial resistance and highlights ZnO NP’s role in overcoming the limitations of EOs, such as low bioavailability and rapid oxidation. Additionally, the potential of these hybrid advanced materials for extending the shelf life of food products, enhancing therapeutic properties, and improving plant disease management is explored. This review provides a comprehensive overview of recent advances, underscoring the potential of ZnO NP and EO systems as advanced materials to address pressing challenges in public health and industry.

## Graphical abstract

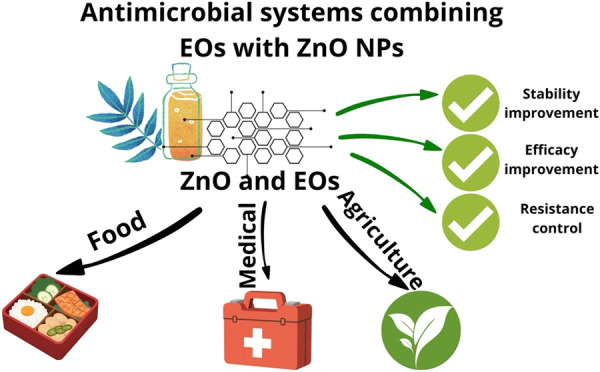

Zinc oxide nanoparticles (ZnO NPs) are multifunctional materials used as safe food additives by the Food and Drug Administration. They are cost-effective semiconductors with excellent chemical stability, biocompatibility, and broad-spectrum antimicrobial properties,^1^ effective against microorganisms like *Escherichia coli*,^2–6^ *Pseudomonas aeruginosa*,^7–12^ and *Staphylococcus aureus*,^11,13–16^ making them suitable for applications in textiles,^17–21^ packaging,^22–26^ and medicine.^27–34^ The ZnO NPs have a high surface area and hydrophilic nature, leading to aggregation, but surface modification can prevent this and improve compatibility with the polymer matrix.^35^ Antimicrobial resistance has become a global threat, developing an urgent need for new treatments to replace existing antibiotics for bacterial infections.^36,37^

Nanotechnology has gained significant attention due to the unique properties of NPs, which are now being utilized in diverse fields, including food processing, agriculture, and medicine.^36,38–40^ Natural antimicrobial agents and metal NPs (copper, silver, selenium, or gold) are added to film matrices to inhibit microbial growth, preserve quality, and extend the shelf life of food and packaged products.^35,41^ Essential oils (EOs) consist of volatile compounds that enhance the physical and structural properties of polymeric matrices.^1,42,43^ The EOs are typically transparent when fresh but may darken with age due to oxidation.^44^ They are extracted through physical processes that minimally alter their chemical composition. These substances are generally hydrophobic, soluble in alcohol and nonpolar solvents,^45^ but only slightly soluble in water, and they are prone to oxidation by light and heat due to the presence of olefinic double bonds and functional groups such as hydroxyl.^44^ Their organic structure provides antimicrobial properties to the matrices.^1^ Herbal EOs (e.g., clove oil) are a preferred natural food preservative due to their safety and excellent functional properties,^46^ making them viable alternatives to synthetic additives in food packaging.^47,48^ Encapsulation of EOs in biopolymer matrices is a promising solution, offering environmental safety and cost-effectiveness.^49,50^ For example, chitosan (CH)-ZnO nanocomposites combined with EOs could enhance the antimicrobial effectiveness of active coatings.^47^ Additionally, despite technological advancements in modern agriculture, managing plant diseases remains challenging due to the limited effectiveness of chemical products like bactericides and fungicides, which often come with negative side effects.^51,52^ The use of plant EOs and nanomaterials has been researched for managing plant diseases, as they are considered safer alternatives to synthetic chemicals.^53^

The aim of this review is to identify advancements in ZnO NPs and EOs systems development, relating to advanced performance evaluation. Integrating ZnO NPs with EOs represents a breakthrough in developing multifunctional products by enhancing the oils’ antimicrobial and antioxidant properties^54–57^ while also improving stability^49,58^ and controlled release.^59,60^

## 1 Synergistic Potential of ZnO NPS and EOS

A significant issue with the overuse of antibiotics is that microorganisms are developing resistance, necessitating unsustainably higher doses.^61,62^ To address this, researchers globally are exploring new antimicrobial options, ranging from innovative synthetic organic compounds,^63–65^ metallic and metal oxide NPs,^66–69^ and natural antimicrobials such as plant extracts or EOs,^69–73^ to synthetic peptides^74–77^ engineered with antibacterial properties compared to antibiotics.^53^ Nanoencapsulation enhances the stability of bioactive compounds (BACs) and enables their controlled release at targeted physiological sites.^78–80^ For food and nutraceutical applications, commonly used materials include carbohydrate-, protein-, or lipid-based options like CH, peptide–CH, and β-lactoglobulin NPs, while BACs such as antioxidants, antimicrobials, vitamins, probiotics, and prebiotics are frequently utilized.^78^ Combining ZnO NPs with EOs enhances antimicrobial effectiveness, either by boosting potency or reducing the required dosage to combat microorganisms. These loaded NPs can be applied in topical ointments and antimicrobial applications such as food packaging.^35,53^ Currently, there is a growing focus on the controlled release of bioactive materials under specific conditions to extend shelf life and optimize nutrient absorption (Table 1). Encapsulation has emerged as a promising technique for protecting sensitive materials from adverse conditions or degradation caused by oxidation.^78^ The EOs incorporated into ZnO NPs can retain volatile components longer and enable the slow release of compounds, improving the availability and sustained antimicrobial activity of the EO components over time.^53^

**Table 1:** Recent Studies of ZnO NPs for EOs Application

**essential oil**	**application field**	**refs**
**wild marjoram, Pune-Sa, white wormwood, and key lime**	Mycelial growth of Alternaria solani, aiming to identify a viable alternative to synthetic chemicals.	^52^
**tea tree**	tissue engineering.	^1^
**wild marjoram**	storage time of refrigerated seafood optimization.	^46^
**clove**	self-life of pork meat improvement.	^45^
	active food packaging.	^35^
**Ylang-ylang**	antibacterial efficacy *in vitro* and *in vivo.*	^36^
**black caraway and aniseed**	3CL protease of SARS-CoV-2 inhibition.	^81^
**Bedda nut tree**	the fertilizing and disease suppression capabilities on *Brassica juncea*.	^51^
**Geraniol**	control of *Botrytis cinerea* in tomato and cucumber plants.	^54^
**Thymus**	shelf life prolongation of sweet cherry.	^82^
	packaging of fresh collard greens.	^83^
	protective role against oxidative stress and cytotoxicity in rats.	^59^
**Lemongrass**	controlled release and enhanced stability of EO.	^48^
**Oregano**	active food packaging.	^84^
**Galbanum**	aquatic packaging industry.	^55^

### 1.1 Antibacterial Effects

Currently, NPs are being increasingly employed as an alternative to antibiotics for controlling bacterial infections.^85–88^ Their use is prevalent in antimicrobial coatings on implantable devices and medical materials^89–93^ to prevent infections and enhance wound healing,^94–99^ as well as in bacterial detection for diagnostics^100–102^ and antibiotic delivery systems.^68,103,104^ Antibacterial composite films are utilized for various applications, including coatings for implants and catheters, as well as wound dressings. Natural polymers like cellulose and collagen are favored for their biocompatibility, while synthetic polymers such as polyethylene, polycaprolactone, and polyurethane are chosen for their durability and ease of processing.^105^ The antimicrobial activity of metal-based NPs follows a multistage mechanism involving initial electrostatic attraction to the bacterial surface, followed by membrane destabilization, increased permeability, and intracellular damage mediated by oxidative stress and signaling disruption.^106^

At the mechanistic level, ZnO NPs act as multifunctional antimicrobial agents where surface reactivity plays a central role in initiating biological interactions. Their high surface energy promotes adhesion to bacterial membranes, facilitating localized oxidative stress and ion-mediated toxicity.^68^ The synergistic interaction between ZnO NPs and EOs arises from the complementary antimicrobial pathways. ZnO NPs primarily induce oxidative stress through ROS generation and Zn^2+^ release,^107,108^ while EOs disrupt microbial cell membranes through hydrophobic interactions with lipid bilayers. This dual-mode action creates a sequential damage mechanism: essential oils first compromise membrane integrity by increasing lipid bilayer fluidity, which enhances nanoparticle attachment and facilitates deeper penetration of Zn^2+^ ions into the cytoplasm. This initial membrane destabilization enhances Zn^2+^ uptake and increases the intracellular susceptibility to ROS-mediated damage (Figure 1). Simultaneously, oxidative stress weakens microbial defense systems, making cells more vulnerable to essential-oil-induced membrane disruption. At the cellular level, this results in a feedback amplification loop, where membrane disruption accelerates oxidative stress accumulation, while ROS further intensifies lipid peroxidation, protein denaturation, and DNA damage. Together, these interactions result in multitarget cellular damage, reducing the likelihood of microbial survival and adaptation.^109^

**Figure 1:**
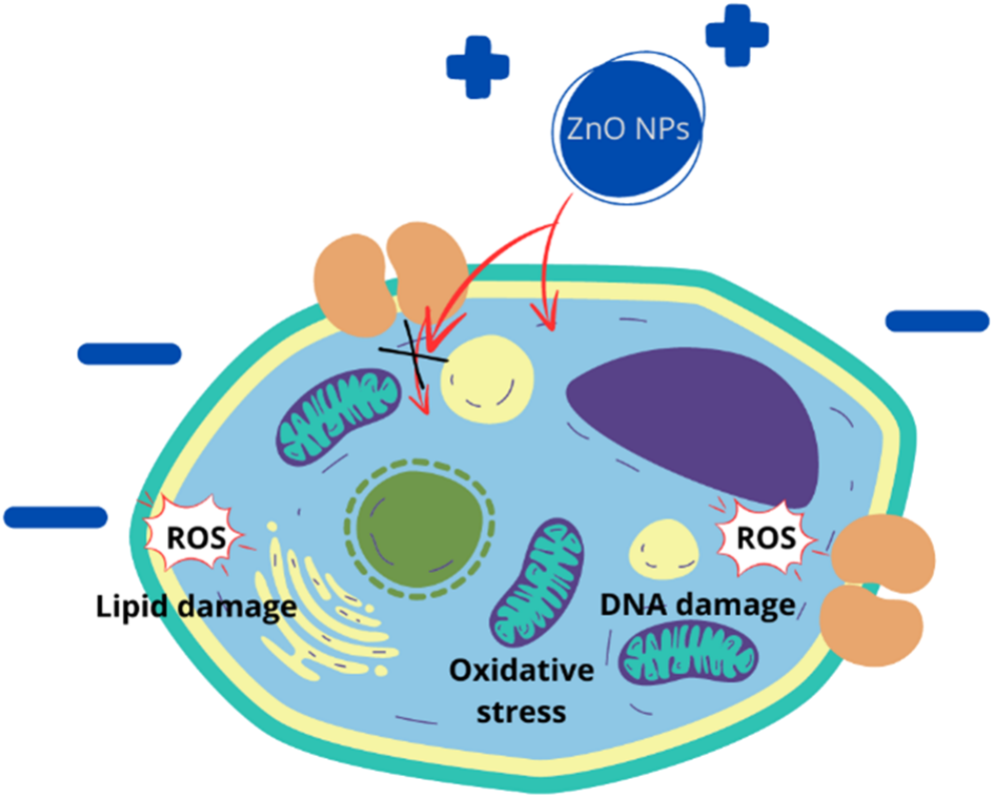
Antibacterial effect of oxidative stress mediated by ZnO NPs.

The magnitude of synergy is not universal and depends strongly on factors such as EOs composition, NPs surface chemistry, and the degree of nanoparticle–lipid membrane interaction. Variability in these parameters can lead to either synergistic, additive, or in some cases negligible effects.^85,110,111^ It is also worth noting that the combined or synergistic effects of multiple EOs may enhance their overall antipathogenic activity. Studies suggest that blending EOs can lead to additive or even synergistic interactions between their bioactive constituents, potentially improving efficacy against a broader spectrum of microorganisms while reducing the likelihood of resistance development.^111^ Such combinations may disrupt microbial cell membranes more effectively or interfere with multiple cellular targets simultaneously.^112^ Consequently, exploring EO compositions rather than single compounds represents a promising direction for developing natural antimicrobial strategies.

## 2 ZnO in Wastewater Treatment

Water scarcity and increasing wastewater generation represent major global challenges, particularly in regions where untreated wastewater is still reused for agricultural irrigation, raising significant environmental and public health concerns.^110^ Wastewater treatment therefore plays a crucial role not only in improving water availability but also in enabling resource recovery,^111,112^ reducing environmental burden, and supporting agricultural productivity through nutrient recycling.^113–115^

Within this context, ZnO NPs have emerged as versatile materials for wastewater remediation because of their combined adsorption capacity, photocatalytic activity, and ease of functional modification. Across multiple studies, ZnO-based systems have demonstrated effectiveness in removing a wide range of contaminants, including heavy metals, dyes, and industrial pollutants; however, performance is highly dependent on the synthesis method, surface modification, and operational conditions.

For heavy metal removal, ZnO nanostructures such as nanorods have shown promising adsorption capacity for arsenic species, with reported spontaneous and thermodynamically favorable uptake processes under near-neutral pH conditions.^116^ Similarly, ZnO-based adsorbents demonstrate strong affinity for toxic metal ions such as Cr­(VI) when incorporated into composite materials (e.g., kaolin/ZnO), where improved surface area and immobilization significantly enhance removal efficiency and reusability.^117^ These findings indicate that ZnO performance is strongly influenced by surface engineering and composite formation rather than intrinsic adsorption alone.

In dye-contaminated wastewater, ZnO NPs exhibit efficient removal of organic pollutants such as synthetic dyes and phenolic compounds, although the adsorption efficiency varies significantly with pH, dosage, and pollutant concentration.^118^ For instance, higher removal efficiencies are generally observed under acidic conditions for dye adsorption, while phenol degradation follows adsorption kinetics consistent with pseudo-second-order models.^119–121^ However, reported efficiencies differ widely between synthetic and real wastewater systems, highlighting a persistent gap between the laboratory and environmental conditions.

Beyond adsorption, ZnO-based materials also function as photocatalysts and catalytic supports in advanced oxidation processes. Composite systems combining ZnO with clay minerals or TiO_2_ significantly enhance pollutant degradation when integrated with ozonation or electroflocculation processes. These hybrid systems consistently outperform single-component ZnO catalysts, achieving near-complete removal of color and high chemical oxygen demand (COD) reduction under optimized operational parameters.^120^ This suggests that ZnO’s catalytic performance is maximized when integrated into multicomponent systems rather than used in isolation.

Green synthesis approaches using plant extracts or microorganisms further expand ZnO applicability by providing environmentally friendly production routes while maintaining adsorption and photocatalytic functionality.^122^ In addition to pollutant removal, treated wastewater from biosynthesized ZnO systems has shown secondary benefits in agriculture, including improved plant physiological responses and reduced oxidative stress markers, indicating potential reuse value.^123^ However, such studies remain limited and largely exploratory.^123,124^

Despite these promising outcomes, several limitations persist across the ZnO-based wastewater treatment systems. First, adsorption performance is highly sensitive to environmental variables such as pH, temperature, and competing ions, which reduce predictability under real wastewater conditions. Second, most studies rely on synthetic effluents, limiting the direct environmental relevance. Third, issues such as NP recovery, long-term stability, and potential secondary contamination are rarely addressed, raising concerns about scalability and environmental safety.^125,126^

Overall, ZnO NPs demonstrate strong potential as multifunctional materials for wastewater remediation, particularly when they are integrated into composite or hybrid systems (Figure 2). However, current evidence indicates that their performance is not intrinsic but highly system-dependent, and future research should focus on improving stability, real-world applicability, and lifecycle safety assessment to enable practical environmental deployment.

**Figure 2:**
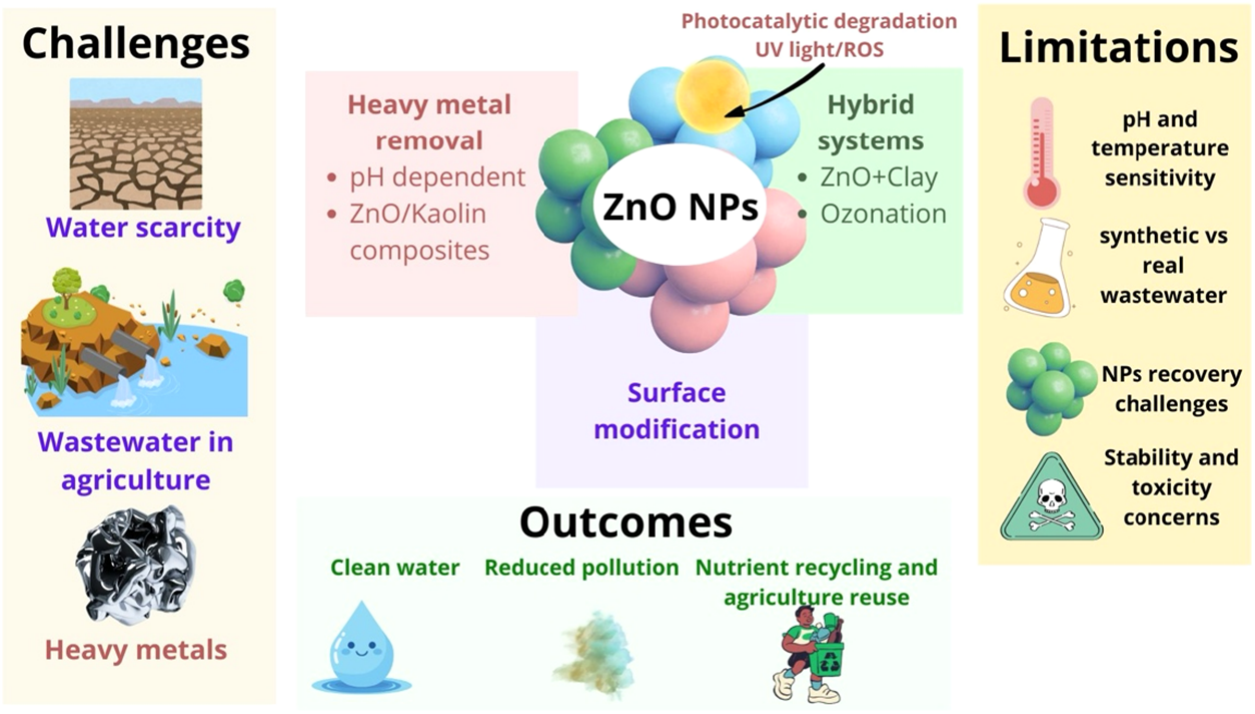
Multifunctional role of ZnO NPs in wastewater treatment and resource recovery.

## 3 Food Packaging

The development of biobased active food packaging films has gained significant attention due to the increasing demand for sustainable, biodegradable alternatives to conventional plastic packaging. These materials are generally nontoxic, biocompatible, and capable of incorporating functional bioactive compounds such as antioxidants and antimicrobial agents without significant risk of harmful chemical migration into food systems.^82^ In this context, ZnO NPs and EOs have emerged as key functional additives for improving the performance of the biodegradable polymer matrices.

Across recent studies, a consistent trend is observed: the incorporation of ZnO NPs—often in combination with EOs and reinforcing agents such as nanoclay, plasticizers, or cross-linkers—significantly enhances the physicochemical and antimicrobial performance of biopolymer-based films. However, the magnitude of improvement strongly depends on polymer type, additive composition, and structural design of the film system.

In chitosan-based systems, multicomponent formulations containing ZnO NPs, CaCl_2_, nanoclay, and plasticizers such as PEG have demonstrated improved postharvest preservation of fruits, including reduced weight loss, lower microbial spoilage, and better retention of biochemical quality parameters.^82^ Similar multiadditive strategies applied to thymus EO-containing films further extend shelf life in leafy vegetables,^83^ suggesting that synergistic combinations rather than single additives provide superior preservation performance in perishable produce.

In protein- and polysaccharide-based matrices, ZnO-based hybrid systems combined with antimicrobial EOs such as clove,^45^ rosemary,^125^ and citronella^127^ consistently improve antimicrobial activity, particularly against foodborne pathogens such as *Staphylococcus aureus* and *Bacillus cereus*. Notably, systems incorporating ZnO NPs with EO-loaded Pickering emulsions demonstrate enhanced lipid oxidation control and microbial suppression in meat products,^45^ indicating that emulsion-based delivery systems improve dispersion and functionality of hydrophobic EO compounds.

Similarly, biodegradable polymer films such as alginate and sodium caseinate exhibit improved barrier and mechanical properties when they are reinforced with ZnO NPs. These enhancements include reduced water vapor permeability, improved UV-blocking capacity, and increased tensile strength, which collectively contribute to better preservation of dairy and cheese products.^128^ Importantly, the addition of EOs alongside ZnO NPs not only enhances antimicrobial activity but also introduces multifunctionality, including antioxidant effects and sensory preservation.^129^

Electrospun nanofiber systems based on polycaprolactone–gelatin matrices further demonstrate that ZnO–EO combinations can be tuned for controlled antimicrobial release.^130^ These systems show improved thermal stability and mechanical performance at optimized nanoparticle concentrations, while maintaining biocompatibility and reducing bacterial growth during refrigerated storage. However, performance is highly concentration-dependent, and excessive nanoparticle loading may negatively affect film flexibility or cellular compatibility.

Overall, while ZnO NPs and EOs consistently improve the antimicrobial performance and functional properties of biodegradable packaging films, the literature reveals substantial variability in outcomes due to differences in formulation strategy, polymer matrix, and processing technique. Moreover, most studies focus on short-term storage and laboratory-scale evaluations with limited assessment under real industrial conditions.

Thus, although ZnO–EO-based packaging systems show strong promise for extending food shelf life and improving food safety, further work is required to optimize formulation standardization, long-term stability, and scalability for commercial applications.

## 4 Drug Delivery Systems

The advancement of novel drug delivery systems (DDSs) has become increasingly important for improving therapeutic precision, safety, and efficacy.^42^ Recent developments focus on integrating advanced materials and nanotechnology to enable targeted and controlled drug release,^131^ addressing key limitations of conventional delivery approaches such as poor solubility, instability, and low bioavailability.

At the same time, natural compounds are gaining attention as promising therapeutic agents for a wide range of diseases, including cancer, diabetes, cardiovascular, and inflammatory disorders,^128–130,132^ due to their reduced toxicity, cost-effectiveness, and diverse biological activities.^132^ However, their broader application remains constrained by challenges in biocompatibility,^132^ compound identification,^129^ limited availability, and difficulties in clinical translation.^132^

To overcome these limitations, emerging DDS strategies increasingly combine natural bioactive compounds with nanomaterials to enhance their stability, delivery efficiency, and therapeutic performance.^131,133–135^ Among these approaches, the incorporation of ZnO NPs with EOs has shown particular promise. This combination not only improves the physicochemical stability of volatile compounds but also enables sustained and controlled release, enhancing antimicrobial efficacy over time.^131,133,136^

Furthermore, recent studies demonstrate that nanostructured composite systems—incorporating polymers, ZnO NPs, and encapsulated EOs—can significantly improve encapsulation efficiency, mechanical properties, and synergistic antimicrobial activity.^53,137,138^ These advances highlight the potential of multifunctional DDS platforms in addressing current challenges associated with natural compounds.

### 4.1 Parameters Identifying Advanced Oil Release Performance

Utilizing essential oils (EOs) loaded onto ZnO nanoparticles (ZnO NPs) enhances key physicochemical properties, including improved retention of volatile components and the controlled release of active compounds. Interactions between EOs and the nanoparticle surface prolong the availability of bioactive constituents, thereby supporting sustained antimicrobial activity and highlighting the potential of this system as an advanced delivery platform (Table 2).^137^ Due to their composition and high volatility, some EOs, particularly those with nonsubstituted terpenes like limonene, demonstrate low loading capacities (e.g., 1.44% for orange EO). In contrast, EOs containing alcohol, aldehyde, and ketone derivatives exhibit higher loading capacities (e.g., 15.62% for cinnamon EO).^137^ For example, Grande-Tovar et al. synthesized four types of PCL-polylactic acid (PLA)­ZnO NPs/glycerol/tea tree EO membranes, showing enhanced thermal stability compared to their pure components, with ZnO NPs particularly improving the thermal degradation and stability of EO. The surface morphology revealed that the presence of tea tree EO and ZnO NPs led to a rough, drop-matrix structure due to polymer incompatibility. Additionally, the presence of EO and ZnO NPs was confirmed by characteristic bands, with the broadening of the C–O–C band indicating intermolecular hydrogen bonding, while the nanocomposites demonstrated biocompatibility in a subdermal implantation model, promoting tissue healing and integration without adverse immune responses.^1^

**Table 2:** Some Examples of EO and ZnO NP Systems’ Thickness and Mechanical Properties

**type of the system**	**thickness**	**mechanical properties**	**refs**
**the chitosan/Melissa EO/ZnO composite film**	the addition of Melissa EO and ZnO NPs significantly affects film thickness (*P* < 0.05). Incorporating both components lead to an increase in thickness, likely due to the intermolecular interactions between chitosan chains, ZnO NPs, and the EO. These interactions enhance film density, contributing to the observed increase in thickness.	Melissa EO significantly reduced the water solubility of chitosan films due to its nonpolar compounds, which limit water molecule interactions (*P* < 0.05). This decrease in solubility is also attributed to the hydrophobic properties of ZnO NPs and the EO, which reduce free hydrogen groups and strengthen the chitosan film structure through enhanced interactions.	^139^
**the gelatin-based films (bovine gelatin/ZnO/clove EO)**	the thickness of neat bovine skin gelatin type-B (BSG) films increased from 0.069 ± 0.001 to 0.073 ± 0.004 with the addition of ZnO and clove EO. This increase in thickness is likely due to the interaction between the protein chains of gelatin and the active compounds in the clove EO.	the TS of neat BSG films decreased significantly (*P* < 0.05) with the addition of 2% ZnO and 25% clove EO, while EAB improved from 15.05 ± 1.3 to 19.1 ± 1.8%. This change in mechanical properties is due to the combined effects of ZnO nanofillers, which typically enhance TS, and the clove EO, which disrupts polymer chain interactions and increases flexibility, leading to reduced TS but improved elongation.	^140^
**ZnO nanorods/clove EO-incorporated** type-B gelatin composite films	the thickness of BSG films increased linearly with the addition of ZnO nanorods and clove EO (*P* < 0.05), which was anticipated due to the higher solid content in the films. The incorporation of these components contributed to the overall increase in film thickness.	this indicates that clove EO functions as a plasticizer in the film matrix, enhancing chain mobility and reducing the strength of intermolecular forces within the protein matrix.	^110,141^
**polylactide/poly(ε-caprolactone)/ZnO/clove EO composite antimicrobial films**	the thickness of the film increased from 0.075 ± 0.001 mm to 0.083 ± 0.001 mm with the addition of ZnO, due to the higher solid content. However, incorporating cloveEO did not affect the film’s thickness.	the addition of clove EO plasticized the films by lubricating their surfaces, leading to a reduction TS to 10.93 MPa and a notable increase in EAB to 204%. However, incorporating both ZnO and clove EO into the films preserved the TS at 13.96 MPa, similar to the ZnO films, while significantly enhancing the EAB to 136.1%.	^110^
**gelatin/agar-based multifunctional film integrated with copper-doped ZnO NPs and clove EO pickering emulsion**	the thickness of the neat gelatin/agar film, initially around 59 μm, increased significantly with the addition of polyethylene carbonate (PEC) and ZnO@Cu, due to the higher solid content pushing the polymer chains apart. Both ZnO@Cu and PEC are thought to interact with the polymer matrix through hydrogen bonding, with ZnO@Cu engaging with the polymer’s hydroxyl groups and PEC interacting with the polymer through its CNF hydroxyl groups.	the TS of the neat gelatin/agar film, initially around 46 MPa, increased to approximately 66 and 55 MPa with the addition of PEC and ZnO@Cu (2 wt %), representing increments of 43% and 19%, respectively. ZnO@Cu enhances TS by forming hydrogen bonds with individual polymer chains, which reinforces the film and improves load distribution due to the alignment of nanoparticles within the polymer matrix.	^45^
**skin gelatin film containing basil leaf EO and ZnO NPs**	the thickness of the films increased with higher levels of basil leaf EO (*p* < 0.05), regardless of the ZnO NPs addition. This increase is likely due to the protruded structures resulting from interactions between EO’s chemical components and the protein matrix, which prevents the formation of a compact and ordered film network, as reflected by the greater thickness.	films containing basil leaf EO exhibited lower TS but higher EAB compared to the control films without EO, regardless of ZnO NP addition (*p* < 0.05). Among all EO-incorporated films, those with 100% EO showed the lowest TS and the highest EAB (*p* < 0.05). The inclusion of EO leads to a more heterogeneous film matrix, causing discontinuities in the film network, which results in reduced rigidity and strength.	^142^
**biodegradable alginate films with ZnO NPs and citronella EO**	a portion of the citronella EO is adsorbed onto the surface of ZnO NPs, while the remainder is dispersed within the film thickness as droplets. As the amount of ZnO NPs increases, more EO is adsorbed onto the NPs, leading to a reduction in the amount of free EO within the film.	the inclusion of ZnO and citronella EO in alginate films has enhanced the light and water barrier properties.	^127^

### 4.2 Stabilization of EO by ZnO against UV and Oxidation Stress

Transparent films play a key role in optics, microelectronics, and related fields, modifying optical properties and providing protection against heat, corrosion, and mechanical wear (Figure 3). However, their performance is highly dependent on deposition conditions, including emission characteristics and substrate positioning, which can lead to nonuniform thickness distribution. This variability directly affects the protective, mechanical, and optical properties of the films,^143^ making precise, rapid measurement of micrometer-scale thickness essential for industrial applications.^139^

**Figure 3:**
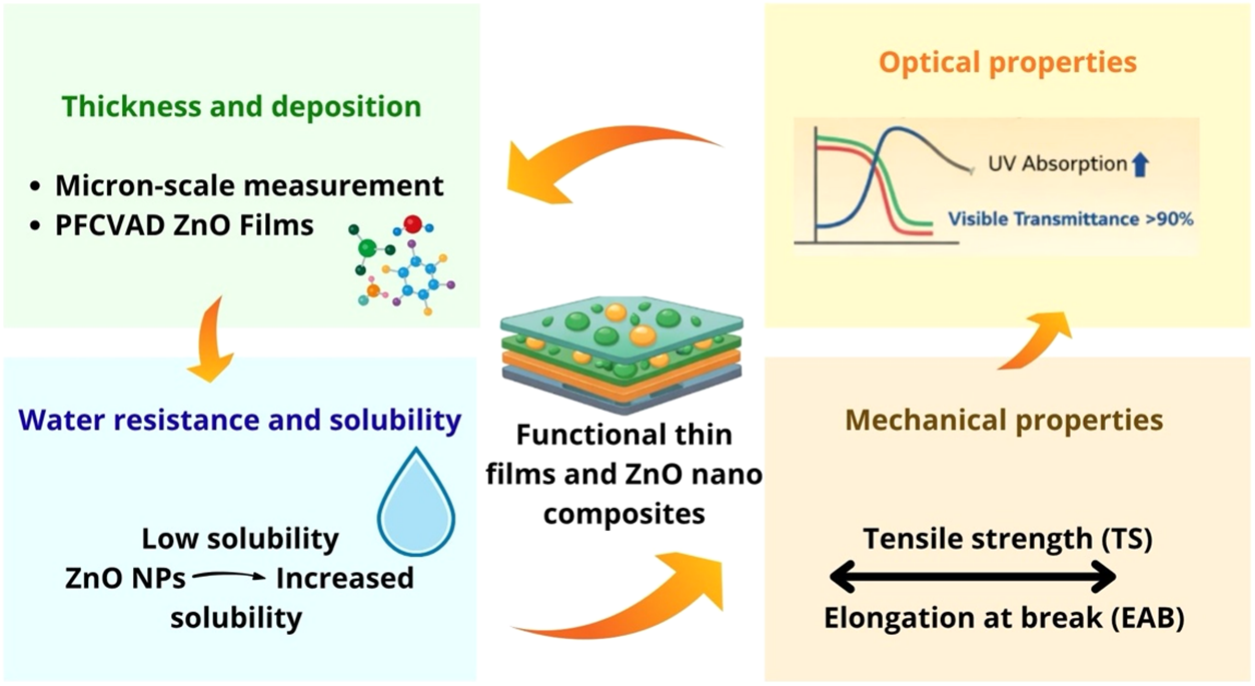
Key physicochemical and functional properties of ZnO-based thin film nanocomposites.

Film thickness is a key parameter influencing both packaging materials and microelectronic components as it determines mechanical integrity, component positioning, and overall operational reliability.^144^ In addition, thickness governs the structural classification of materials into thin films, thick films, or bulk forms, each exhibiting distinct characteristics such as amorphous, polycrystalline, or crystalline structures.^145^ For biodegradable films, additives such as glycerol are commonly used as plasticizers to enhance flexibility, while reinforcement with ZnO NPs further improves mechanical strength and barrier properties through cross-linking within the polymer matrix.^127^ The influence of thickness on material properties is particularly evident in ZnO films deposited using a pulsed filtered cathodic vacuum arc (PFCVAD). These films exhibit a hexagonal wurtzite structure with strong *c*-axis orientation, and increasing the thickness leads to improved crystallinity and larger crystallite size. While visible light transmittance remains above 90%, an increased thickness reduces UV transmission and decreases the optical band gap. Simultaneously, parameters such as refractive index and dielectric constants are altered, demonstrating that film thickness can be used to tune optical properties effectively.^146^

Another critical parameter for film performance is solubility, which reflects water resistance and hydrophilicity.^139^ This property is particularly important for edible films used in food preservation, where excessive solubility can compromise the barrier function by allowing moisture and compounds to diffuse into the food. However, in some applications, controlled solubility prior to consumption may be beneficial.^147^ Studies have shown that incorporating ZnO NPs into polymer matrices reduces solubility by enhancing intermolecular hydrogen bonding and limiting water interaction,^147,148^ whereas certain EOs may increase solubility by weakening these interactions.^149^

Mechanical properties, particularly elongation at break (EAB) and tensile strength (TS), are also essential for evaluating film performance.^150^ EAB reflects the material’s ductility and resistance to deformation, while TS indicates the maximum stress the material can withstand before failure.^151^ Typically, materials with high TS exhibit lower EAB and vice versa.^152,153^ The incorporation of ZnO NPs can significantly influence these properties: at low concentrations, well-dispersed nanoparticles improve interfacial interactions and delay mechanical failure, whereas higher concentrations may lead to stress concentration and reduced mechanical performance.^154^

## 5 Advanced Sensor Development and Performance Analysis

Zinc oxide has emerged as a pivotal material in the field of sensor development due to its unique optical and electrochemical properties.^155–161^ Its photoluminescence (PL) characteristics make it especially suitable for applications in room-temperature sensors, allowing it to serve as a selective analytical transducer when appropriately functionalized.^155–157^ The ability to fine-tune these properties ensures optimal performance for specific sensing environments, thereby enhancing detection sensitivity and signal-to-noise ratios.^155–161^ Various techniques, including electrochemical impedance spectroscopy (EIS), are employed to investigate high-resistance materials like ZnO, offering a noninvasive method to collect quantitative data.^162–164^ The EIS is a highly effective technique with novel application areas^165^ for analyzing interfacial properties associated with biorecognition events at the electrode surface, such as antibody–antigen interactions, substrate-enzyme reactions, or the capture of whole cells.^162,166^

### 5.1 ZnO as Oil Sensors inside the Encapsulation Oil

ZnO-based nanomaterials have been extensively explored in biosensing, gas sensing, oil monitoring, energy, and environmental applications because of their tunable optical, electrical, and surface properties. Surface functionalization strategies, such as polydopamine coating on ZnO nanorods, have been shown to effectively modify electrochemical behavior and enhance biomolecular interactions, enabling improved performance in biosensing platforms.^167^ Similarly, ZnO nanostructures have been widely employed in immunosensors and optical biosensors^164^ for the detection of biomolecules and viruses, including bovine leukemia virus proteins,^156^ prostate-specific antigen,^168^ SARS-CoV-2 antibodies,^157^ and plant viruses,^161^ achieving high sensitivity, rapid response times, and low detection limits through mechanisms based on photoluminescence, impedance changes, and whispering gallery mode resonance.

In gas-sensing applications, ZnO-based nanocomposites and heterostructures (e.g., ZnO/PANI,^155^ ZnO/Ag,^169^ and metal-doped ZnO^170^) have demonstrated efficient detection of volatile organic compounds and dissolved gases in transformer oil, with improved selectivity and sensitivity attributed to enhanced surface area, defect engineering, and catalytic activity. These systems have also been integrated into optical fiber- and SAW-based devices, enabling real-time monitoring of oil quality, water content, and gas composition in industrial systems.

ZnO nanomaterials further play a key role in oil-related applications, including enhanced oil recovery, where ZnO-based nanofluids and ZnO–Ce composites improve interfacial properties and oil displacement efficiency, as well as oil sensing and reservoir monitoring through fluorescent tracers and encapsulated nanostructures. In parallel, ZnO-based photocatalytic and membrane systems have been applied for oil–water separation and environmental remediation, achieving high permeability, strong antifouling behavior, and excellent separation efficiency.

In tribological systems, ZnO NPs and their composites enhance lubricant performance by reducing friction and wear while improving thermal stability and tribofilm formation, particularly when combined with graphene^171^ or optimized dispersion techniques.^172^ Additional studies also highlight their role in catalytic biodegradation of petroleum contaminants when used in combination with microbial systems, achieving rapid and high-efficiency oil breakdown.^173–177^

### 5.2 ZnO NPs in Diverse Applications

Nanoparticles, owing to their nanoscale dimensions and their ability to tune interfacial tension, contact angle, and viscosity, have attracted considerable attention in enhanced oil recovery (EOR) applications.^178–181^ This study evaluated a newly developed ZnO–cerium N-composite under reservoir conditions and compared its performance with standalone ZnO nanoparticles. At an optimum concentration of 100 ppm, the ZnO–Ce composite demonstrated superior stability, enhanced capillary reduction, and improved interfacial properties, including an interfacial tension of 22.51 mN/m, contact angle of 40.83°, and zeta potential of – 44.36 mV, resulting in a significantly higher recovery factor of 71.40% compared to 37.11% for ZnO NPs.^178^

In tribological applications, *Euphorbia lathyris* oil modified via epoxidation and enriched with ZnO NPs exhibited improved lubricant properties, including increased viscosity and flash point, alongside a reduced pour point. Pin-on-disc testing revealed that a 0.5% ZnO NP concentration reduced the coefficient of friction and wear by 8.23% and 5.13%, respectively, with scanning electron microscopy confirming improved wear surfaces.^182^ Similarly, ZnO nanomaterials in commercial engine oils showed synergistic tribological and antioxidant effects, strongly influenced by nanoparticle size, functionalization, and concentration.^183^ In polyester oil nanodispersions, ball milling reduced nanoparticle size from 40 to 30 nm, leading to improved dispersion stability, lower friction coefficients, and enhanced thermal conductivity as measured by the transient hot-wire method.^184^

Beyond lubricants, ZnO NPs have been effectively incorporated into biodegradable and functional materials. In active food packaging, ZnO-based films enhanced antimicrobial and antioxidant performance while reducing moisture absorption, solubility, and vapor permeability, thereby improving the storage quality of meat products at 4 °C through lower pH, reduced volatile nitrogen content, and decreased bacterial growth.^185^

In conservation and protective coatings, ZnO NPs embedded in bioactive films, cellulose-based matrices, and silica coatings demonstrated strong resistance to microbial degradation, UV aging, and environmental contamination while maintaining acceptable optical properties.^186^ Coatings containing ZnO improved the durability of oil paintings, reduced microbial colonization, and facilitated surface cleaning. In anticorrosion applications, silica-based nanocomposites incorporating ZnO and benzotriazole exhibited corrosion protection efficiencies comparable to commercial Incralac coatings, with corrosion rates between 0.044 and 0.067 mm/year.^187^ Additionally, ZnO–cellulose nanocomposites showed uniform nanoparticle dispersion, enhanced color stability under aging, and effective antibacterial and antifungal activity against common archival microorganisms.^188^

Overall, these findings demonstrate that ZnO nanoparticles exhibit broad multifunctionality across energy, tribological, biomedical, packaging, and conservation-related applications. Their performance is strongly dependent on the concentration, dispersion quality, particle size, and composite formulation. This highlights ZnO-based systems as highly adaptable nanomaterial platforms while also emphasizing the need for careful optimization to balance functional performance with long-term stability and application-specific requirements.

## 6 Discussion

A key limitation in the current development of ZnO NPs–EO systems is the limited translation from promising *in vitro* results to clinically or industrially relevant applications. While many studies demonstrate strong antimicrobial efficacy under controlled laboratory conditions, these results do not necessarily reflect performance in complex biological or real-world environments, where factors such as protein corona formation, pH variation, ionic strength, and competing biomolecules can significantly alter nanoparticle behavior and bioactivity.

From a safety perspective, ZnO NPs raise important toxicity concerns related to dose-dependent cytotoxicity, oxidative stress induction in mammalian cells, and potential accumulation in tissues following prolonged exposure.^189,190^ Similarly, EOs, despite their natural origin, may exhibit cytotoxic or irritant effects at higher concentrations, and their rapid volatility further complicates controlled dosing. When combined in hybrid systems, the toxicological profile may not be simply additive, and potential synergistic toxicity effects remain insufficiently explored.

Regulatory translation also represents a major challenge, as standardized guidelines for nano-enabled antimicrobial systems are still evolving. Variability in NPs synthesis methods, surface modifications, and EOs compositions leads to inconsistent material properties, making reproducibility and regulatory approval difficult. Furthermore, the absence of standardized toxicity testing protocols for ZnO–EO hybrid systems limits their comparability across studies and hinders progress toward clinical validation.

## 7 Future Perspectives

As the demand for waste reduction and the pursuit of sustainable development goals intensify, there is a need to adopt green chemistry principles in developing alternative, eco-friendly methods for NP synthesis.^191–198^ Among metal oxide NPs, ZnO NPs are notable for their unique properties, such as large excitation binding energy and a wide, direct band gap, making them useful in various biological applications like drug delivery, biosensing, and antibacterial activity. Traditional physical and chemical synthesis methods for ZnO NPs involve high-end instruments and toxic chemicals, making them costly and less eco-friendly, whereas green synthesis using phytoproducts offers a more sustainable and advantageous alternative.^36^

Naiel et al. described the effectiveness of using the aqueous extract of sea lavender for the green synthesis of hexagonal/cubic ZnO NPs, showing significant anticancer activity against A-431 cells while maintaining biocompatibility with normal cells (WI-38). Additionally, these NPs demonstrated notable antimicrobial and antioxidant properties, underscoring the importance of conserving salt marsh ecosystems for both their environmental benefits and their potential as sustainable sources for nanomaterial synthesis.^191^ Abdelbaky et al. used Cymbopogon citratus aqueous leaf extract to produce ZnO NPs in a green way. The biosynthesized ZnO NPs demonstrated significant selective cytotoxicity toward all tested cancer cell lines while leaving normal cells unaffected. Conversely, the aqueous leaf extract of *C. citratus* had no impact on the cancer cell lines at any concentration tested.^199^

Velumani et al. demonstrated a rapid, cost-effective, and eco-friendly method for synthesizing ZnO NPs using EO from ylang-ylang flowers. The green-synthesized ZnO NPs showed strong antibacterial activity against *Pseudomonas aeruginosa* and *S. aureus* and were nontoxic up to 1 g/L in *Caenorhabditis elegans*, where they also reduced bacterial colonization and enhanced physiological functions. The findings indicate that ylang–ylang EO-mediated ZnO NPs could serve as effective bactericidal agents to combat antimicrobial resistance and improve food packaging technology, especially for ready-to-eat products.^36^

Another eco-friendly method to synthesize ZnO NPs is to use aqueous fruit extracts of the medicinal plant *Myristica fragrans*. The crystalline ZnO NPs demonstrated antioxidant properties, antibacterial activity, and moderate inhibition of α-amylase and α-glucosidase enzymes. Also, NPs showed significant potency against brine shrimps and *Aedes aegypti* larvae, effective methylene blue dye degradation, and biocompatibility with human red blood cells.^200^ One more research proposed Eucalyptus globulus EO application for the green synthesis of ZnO NPs. The EO, extracted from leaves through hydrodistillation, was combined with zinc acetate dihydrate to produce ZnO NPs. The results showed that the ZnO NPs have potent antimicrobial activity against all tested microorganisms, with a maximum inhibition zone of 19.35 ± 0.45 mm for *Klebsiella pneumoniae* at a concentration of 100 μg/mL. Additionally, there was a significant biofilm inhibition of 85% for *S. aureus* ATCC 25923 and 97% for *P. aeruginosa* ATCC 27853.^201^

There are more studies that identify opportunities to synthesize ZnO NPs while using EOs from *Acacia catechu*, *Artemisia vulgaris*, and *Cynodon dactylon*,^179^ *Zingiber officinale* and *Allium sativum*,^180^ *Pelargonium odoratissimum*,^181^ *Nelumbo nucifera*,^182^ and *Salvia Rosmarinus* extract,^183^ geranium,^184^ and lemongrass EOs (Figure 4).^132^

**Figure 4:**
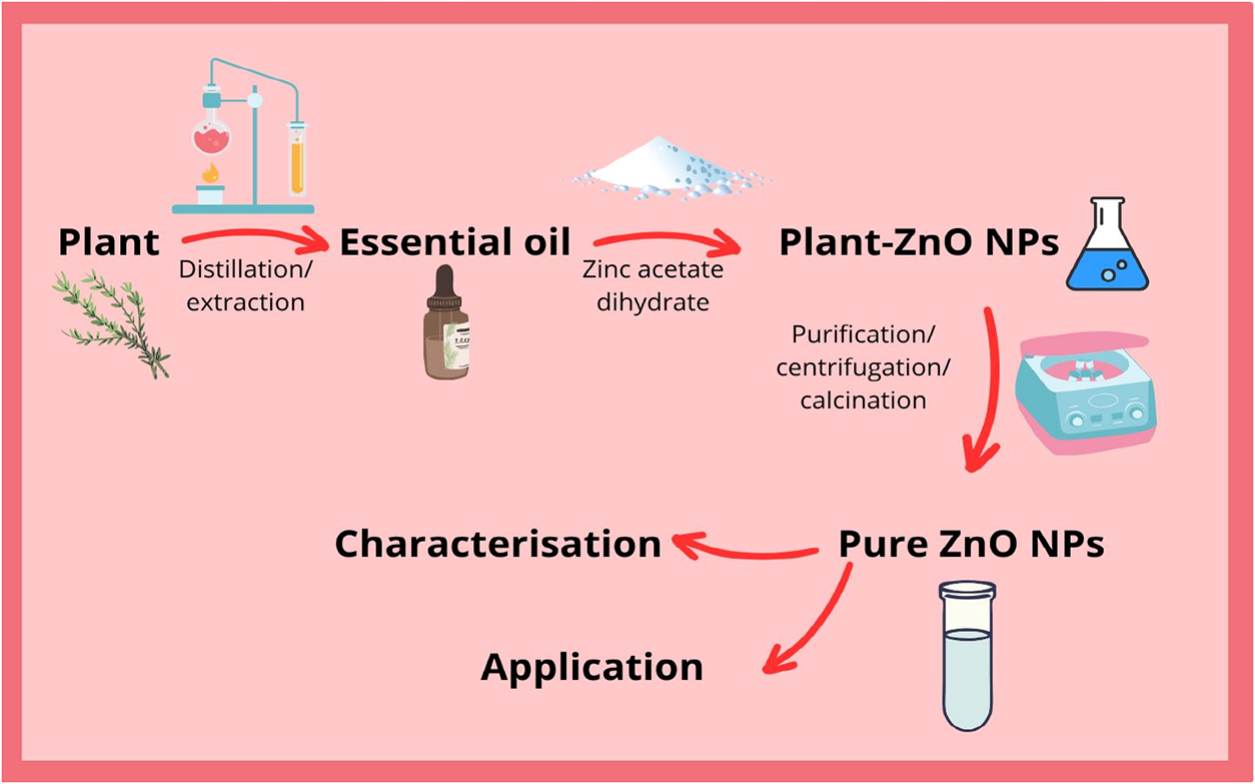
Schematic representation of EOs application in ZnO NPs green synthesis.

The green synthesis of ZnO NPs using EOs presents numerous advantages and holds significant potential for transformative innovations in biomedical applications in the future.^185^ This eco-friendly and sustainable approach eliminates harmful chemicals, reducing environmental impact.^186,187^ The resulting NPs are biocompatible, making them safer for biomedical applications like wound healing and drug delivery.^187,188,202^ The phytochemicals in EOs also stabilize the NPs, enhancing their functional properties, such as drug loading and antimicrobial effectiveness.^203,204^ To advance their practical implementation, green-synthesized ZnO NPs should be integrated into engineered drug delivery systems designed for targeted and controlled therapeutic delivery, thereby minimizing systemic side effects and improving treatment precision. However, despite their promising *in vitro* and early *in vivo* performance, a critical challenge remains the lack of comprehensive evaluation of their biocompatibility, biodegradability, pharmacokinetics, and immunological responses in complex biological environments. This gap between laboratory findings and real-world biomedical application represents a major barrier to clinical translation. Therefore, future research must prioritize systematic *in vivo* validation, long-term safety assessment, and standardized toxicity evaluation protocols to ensure the safe and effective use of green-synthesized ZnO NPs in practical applications.

## 8 Conclusions

In conclusion, the interaction between ZnO NPs and EOs represents a significant advancement in the development of multifunctional products across various sectors. This innovative approach not only enhances the inherent properties of EOs, such as antimicrobial and antioxidant activities, but also provides additional benefits, such as improved stability, increased bioavailability, and controlled release. As research continues to explore and optimize these synergies, the potential for ZnO-NP-enhanced EOs in therapeutic, cosmetic, and preservative formulations becomes increasingly apparent. This promising field offers a sustainable and natural alternative to conventional products, aligning with the growing consumer demand for safer and more effective solutions. As the demand for waste reduction and sustainable development intensifies, adopting green chemistry for NP synthesis, particularly using phytoproducts, presents an eco-friendly and more cost-effective alternative to traditional methods.

## Abbreviations

ALD: atomic layer deposition
BAC: bioactive compounds
BLV: bovine leukemia virus
BSG: bovine skin gelatin type-B
CH: chitosan
COD: chemical oxygen demand
CVD: chemical vapor deposition
DDS: drug delivery systems
DFT: density functional theory
EAB: elongation at break
EOs: essential oils
EOR: enhanced oil recovery
EIS: electrochemical impedance spectroscopy
FTO: fluorine-doped tin oxide
GVA: grapevine virus A-type
HEC: hydroxyethyl cellulose
IZO: in-doped ZnO
MCM-41: mesoporous silica
MIP-Ppy: molecularly imprinted polypyrrole
NBE: near band emission
NCs: nanocrystals
NPDP: ZnO nanoparticles dispersed in polyester oil
NPs: nanoparticles
NR: nanorod
PAH: poly­(allylamine hydrochloride)
PAO 6: polyalphaolefin
PDA: polydopamine
PEC: polyethylene carbonate
PEG: polyethylene glycol
PFCVAD: pulsed filtered cathodic vacuum arc
PLA: polylactic acid
PL: photoluminescence
POF: plastic optical fiber
PSA: prostate-specific antigen
QDs: quantum dots
RF: recovery factor
ROS: reactive oxygen species
SAW: surface acoustic wave
TIRE: total internal reflection ellipsometry
TS: tensile strength
WGM: whispering gallery mode
ZnO: zinc oxide
